# P364: Injection safety and medical waste management in health care institutions in Guinea. (Guinea Conakry)

**DOI:** 10.1186/2047-2994-2-S1-P364

**Published:** 2013-06-20

**Authors:** D Malal

**Affiliations:** 1 Universty of Conakry, Conakry, Guinea

## Objectives

This study of the application of quality standards in the process of seeking care aims to contribute to improving the safety of injections, dressings and biomedical waste management in health institutions.

## Methods

This is a descriptive study on the cross-device management services offered in nursing health facilities. The technique used for data collection is that of Interview. It ran from a questionnaire on the device provides nursing care in nine health facilities targets. This is a sample having a size of 52 subjects distributed among the strata according to their size represented by the staff working in maternity services health facilities drawn.

## Results

The main results are:

i) The professional nursing is the most dominant with 34.59%, ii) Approximately 20% of respondents are on the verge of retirement, and 15% over 50 years, iii) The ability to recognize the workers through their work clothes is very low, iv) There is a weakness in the procedures for dispensation injections and dressings, v) Inadequate hand washing device (38%), vi) Low waste identification based on garbage bags and transportation inadequate, vii) Disinfection of medical devices violating procedures, viii) Low availability of clean cloth, ix) Acts and treatment protocols used infrequently, and x) A third of health professionals do not have standards for the prevention of infectious risk.

## Conclusion

Renew the number of agents maternity; Train staff in the use of care protocols; Improve water availability, garbage bags and disinfection equipment, injection materials and dressing; Strengthen individual protection measures.

## Disclosure of interest

None declared

